# The Dilemma in the Management of Thromboembolic Disease in the Setting of Concomitant Aortic Pseudoaneurysm

**DOI:** 10.7759/cureus.20668

**Published:** 2021-12-24

**Authors:** Anthony Lyonga Ngonge, Nitheesha Ganta, Abdelawab Jalal Eldin, Valery Effoe, Nso Nso, Deborah Williams

**Affiliations:** 1 Internal Medicine, Howard University Hospital, Washington, USA; 2 Cardiology, Howard University Hospital, Washington, USA; 3 Cardiovascular Disease, Morehouse School of Medicine, Atlanta, USA; 4 Department of Medicine, Icahn School of Medicine at Mount Sinai, New York City, USA; 5 Department of Medicine, NYC Health + Hospitals/Queens, New York City, USA

**Keywords:** therapeutic anticoagulation, non valvular atrial fibrillation, stroke, acute pulmonary embolism, aortic pseudoaneurysm

## Abstract

Ascending aortic pseudoaneurysm (AAP) is a rare and serious complication of cardiothoracic surgeries or blunt chest trauma. We present a patient with paroxysmal atrial fibrillation, acute right pontine stroke, and acute pulmonary embolism (PE) with an incidental AAP that precluded the use of anticoagulation and surgery. The case findings substantiate the need for a CT-based assessment of aortic pathology after coronary artery bypass grafting (CABG) in the asymptomatic patient to determine the most appropriate treatment modalities. However, the high cost of CT imaging and the potential radiation exposure challenge its routine use in high-risk patients.

## Introduction

Complex cardiothoracic surgeries rarely lead to the development of ascending aortic pseudoaneurysm (AAP), which is a fatal and life-threatening complication. AAP assessment depends on the concomitant use of diagnostic measures, including ascending aortography, coronary angiography, and CT angiography [1]. AAP has the potential to erode bony structures inside the chest. Suppose it develops in the anterior aortic sinus. In that case, it may manifest as a mass effect under the retrosternal region, which can distort the anatomy of the medial upper chest, sternum, and the main pulmonary artery [2]. Therefore, careful preoperative planning is needed for the surgical correction of AAP. The use of off-label equipment is also recommended for endovascular AAP closure in high-risk surgery patients [3]. AAP also develops from the type-3 aortic injury from high-speed motor vehicle crashes or falls [4]. The clinical signs include pseudocoarctation, diminished femoral pulses, and upper extremity hypertension. Other potential causes of AAP include vascular bypass, degenerative atherosclerotic penetrating lesions, infections, and blunt trauma [5]. The non-surgical interventions for AAP management include liquid/coil embolic agents, septal defect occlusion devices, and modified stent grafts administered via endovascular and transthoracic approaches [6]. In addition, recent research confirms a surgical approach based on the multi-fenestrated cribriform septal occluder implantation for high-risk patients [7].

The routine medical care for managing paroxysmal atrial fibrillation, acute right pontine stroke, acute pulmonary embolism (PE), and myocardial infarction relies on anticoagulants (including warfarin and heparin) and thrombolytic therapy (including tenecteplase, reteplase, urokinase, and streptokinase) [8]. The degenerative vascular pathology of AAP also increases the risk of its spontaneous rupture that may lead to sudden death [9]. The use of anticoagulants or thrombolytic therapy further aggravates the risk of AAP dissection/rupture followed by hypovolemic shock, cardiac tamponade, hemopericardium, and sudden cardiac death [10]. However, exclusion of anticoagulants and thrombolytic therapies in such cases increases the risk of thromboembolic events to many folds.

## Case presentation

We report the case of a 73-year-old female who presented to the ER with left-sided body weakness of unclear duration. She had an ischemic stroke four years prior with no residual neurologic deficits, a myocardial infarction requiring coronary artery bypass grafting (CABG) two years prior, hypertension, and dementia. Her vital signs were blood pressure (BP) 117/78 mmHg, pulse 121 beats per minute, temperature 98.9 F, respiratory rate (RR) 18 cycles/minute, and oxygen saturation (SpO2) of 97% on ambient air. She was disoriented to place and time with a Glasgow Coma Score (GCS) of 14 (E4V4M6). Her speech was slurred, cranial nerves (CN) 2-12 were grossly intact, motor strength on the left upper and lower extremities was 0/5 and on the right upper and lower extremities was 4/5, and the sensation was preserved in all extremities. The patient had a National Institutes of Health Stroke Scale (NIHSS) score of 16 and a Modified Rankin Score (mRS) of 5 points.

**Figure 1:**
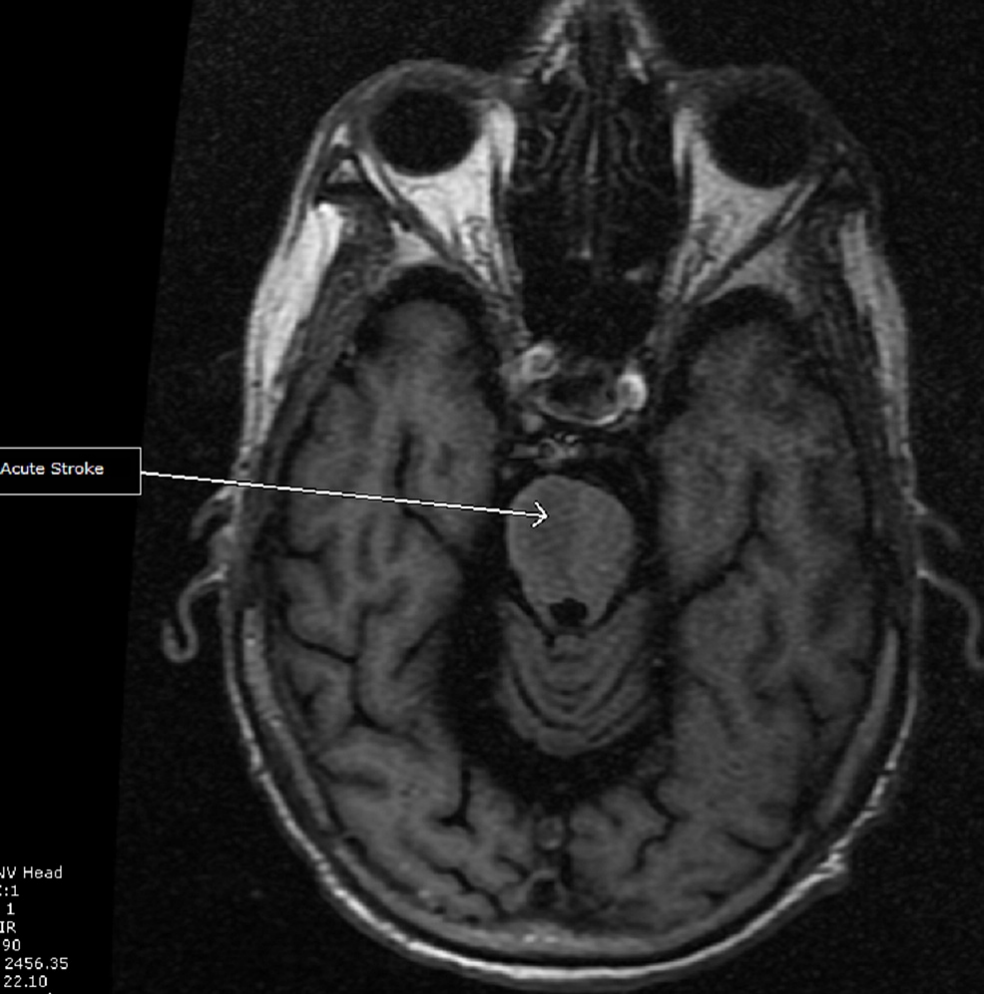
MRI T1 FLAIR with acute pontine stroke.

**Figure 2:**
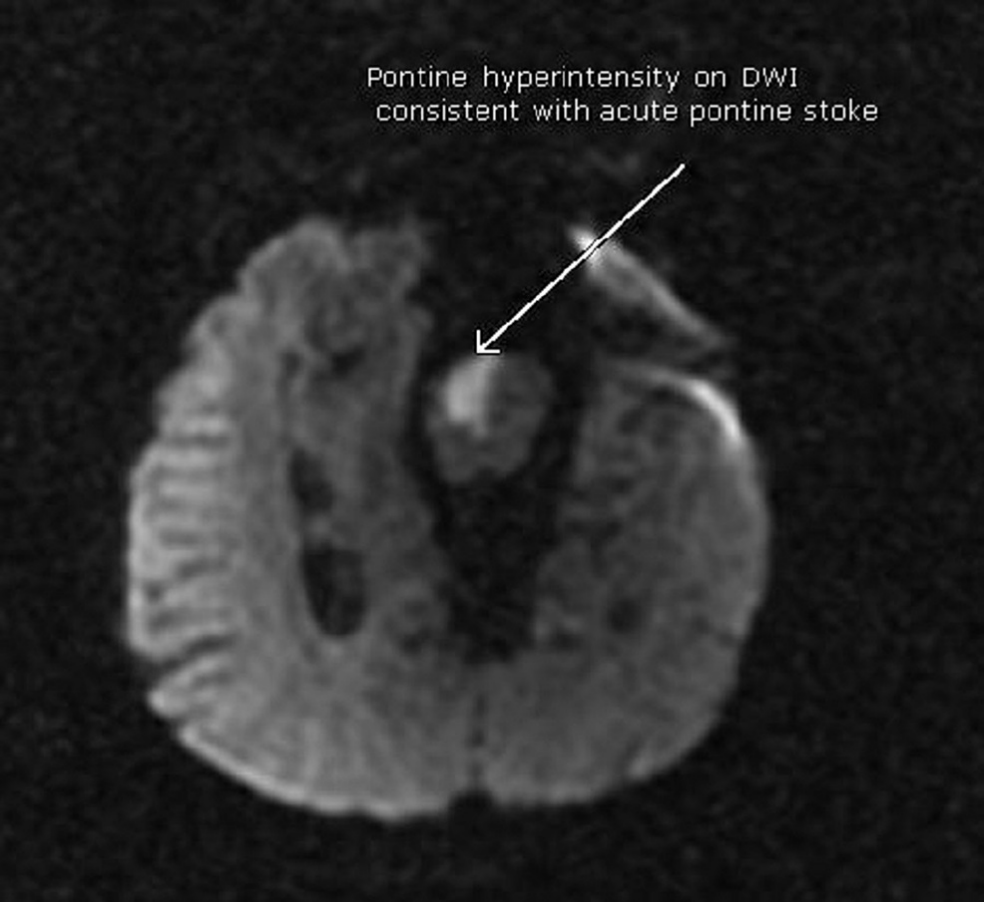
Diffusion-weighted imaging window demonstrating acute pontine stroke.

**Figure 3:**
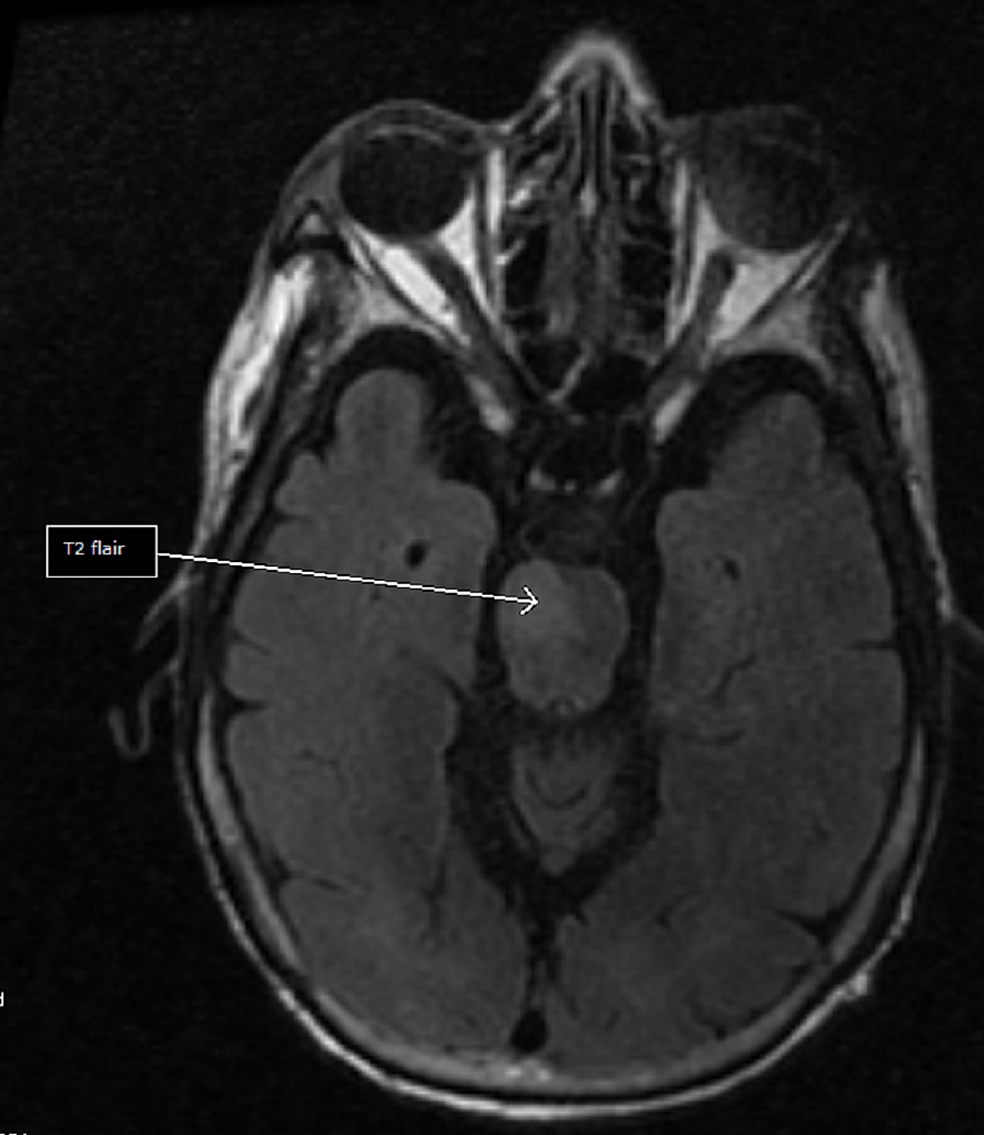
T2 FLAIR MRI imaging. FLAIR: Fluid attenuated inversion recovery.

**Figure 4:**
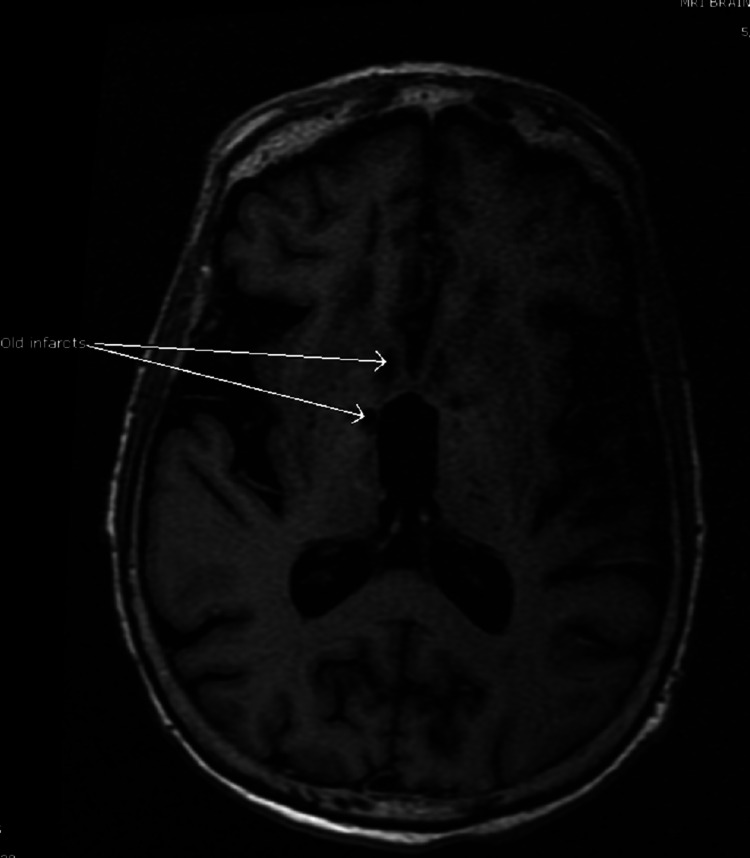
Bilateral old lacuna infarcts on axial T1.

**Figure 5:**
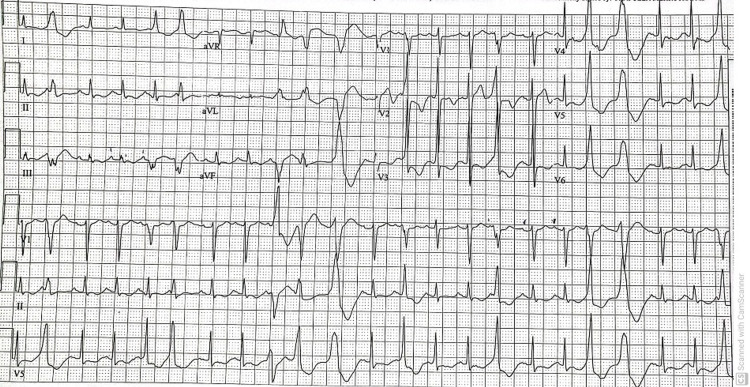
Sinus tachycardia with PVCs and PACs. PVCs: Premature ventricular contractions; PACs: Premature atrial contractions.

A non-contrast head CT scan revealed evidence of old lacuna infarcts in the basal ganglia and thalamus. No intracranial hemorrhage or acute infarct was found. CT perfusion was not done as our center lacks the resources needed to perform that. A brain MRI scan showed an acute pontine stroke (Figures 1-3) and old infarcts (Figure 4). A magnetic resonance angiogram of the neck showed patent internal and external carotid vessels. Initial ECG showed sinus tachycardia with multiple premature ventricular contractions (PVCs) and nonspecific ST-T wave changes (Figure 5). Serum electrolytes, blood urea nitrogen, creatinine, troponin, and glycosylated hemoglobin (HbA1c) were within normal limits.

Given the unknown time of symptom onset, the patient was admitted to the neurology service and started on a non-tPA protocol for stroke management with aspirin, high-intensity statin, and physical therapy. She was also placed on telemetry monitoring, intermittent pneumatic compressive devices, and low dose heparin for deep venous thrombosis (DVT) prophylaxis. Transthoracic echocardiography showed a normal left ventricular function ejection fraction of 55-60%, normal right ventricular function, and normal chamber sizes. No intra-cardiac masses and atrial septal defect (ASD) were noted. 

**Figure 6:**
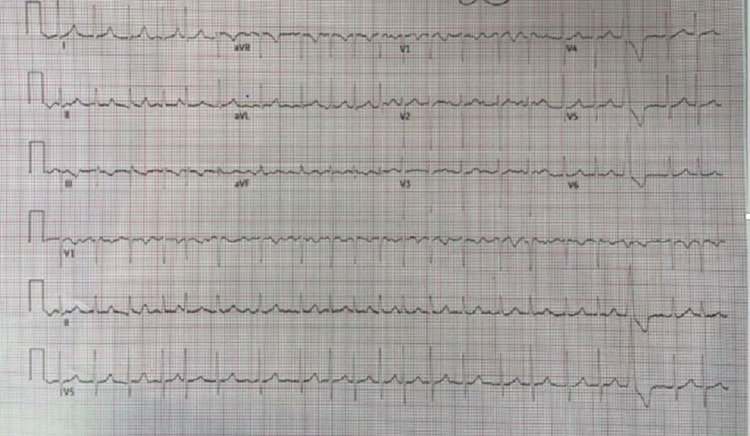
Atrial fibrillation with rapid ventricular response.

**Figure 7:**
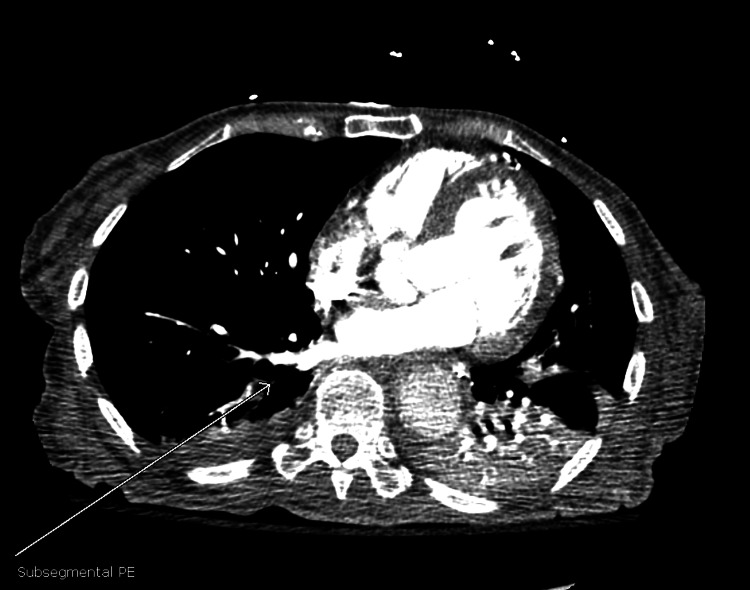
Bilateral sub-segmental pulmonary embolisms.

**Figure 8:**
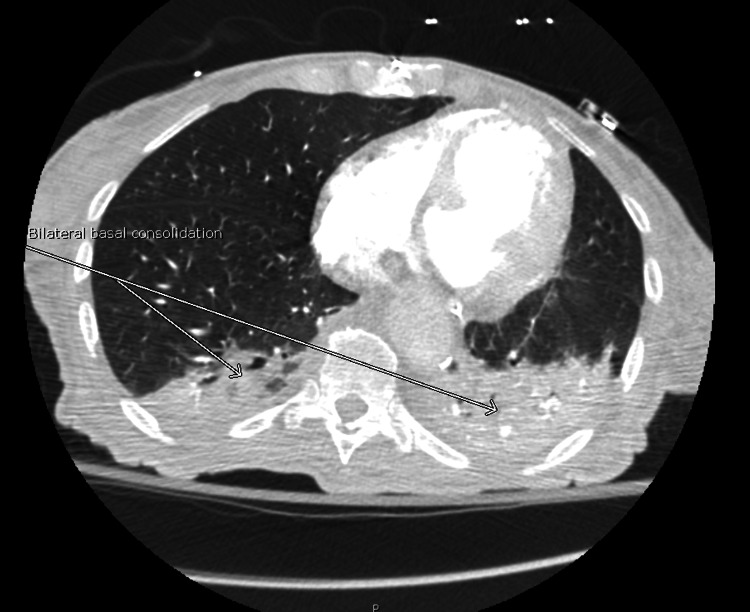
Bilateral lower lung lobe consolidation.

**Figure 9:**
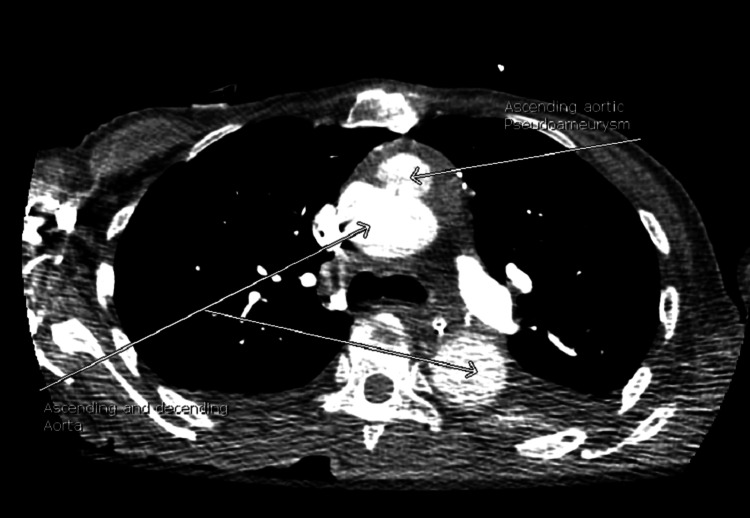
CT angiogram chest depicts ascending aortic pseudoaneurysm.

**Figure 10:**
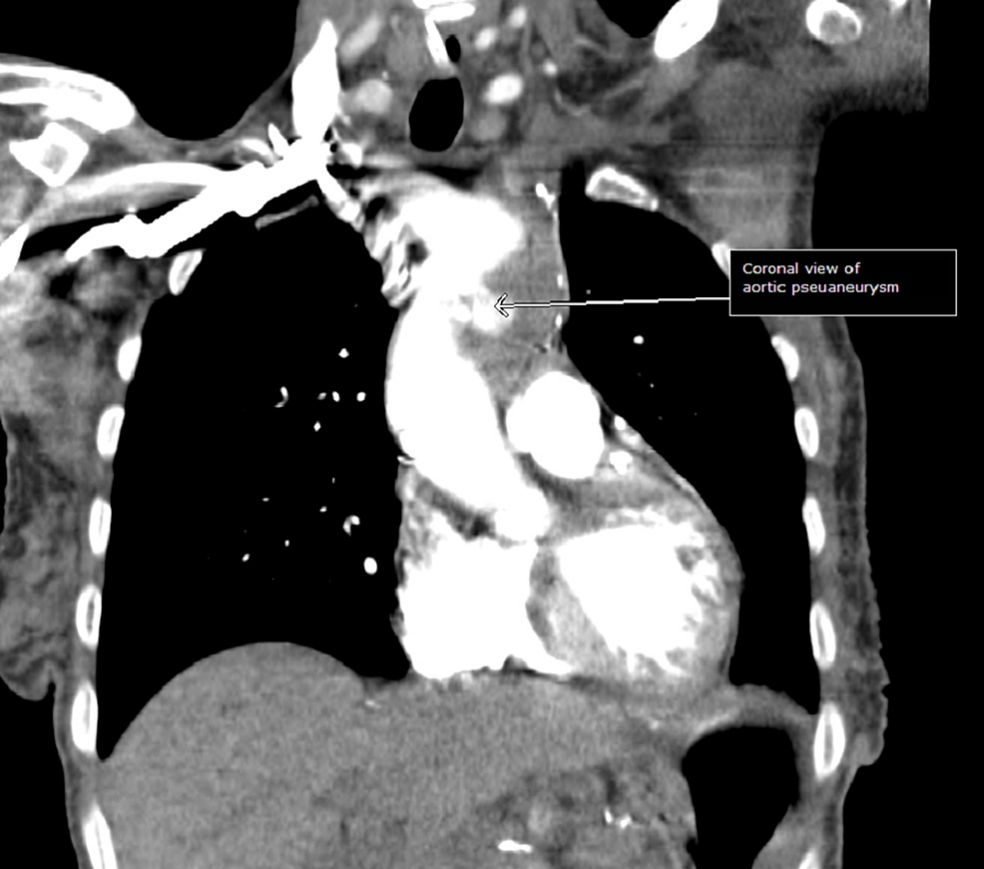
CT angiogram coronal view.

**Figure 11:**
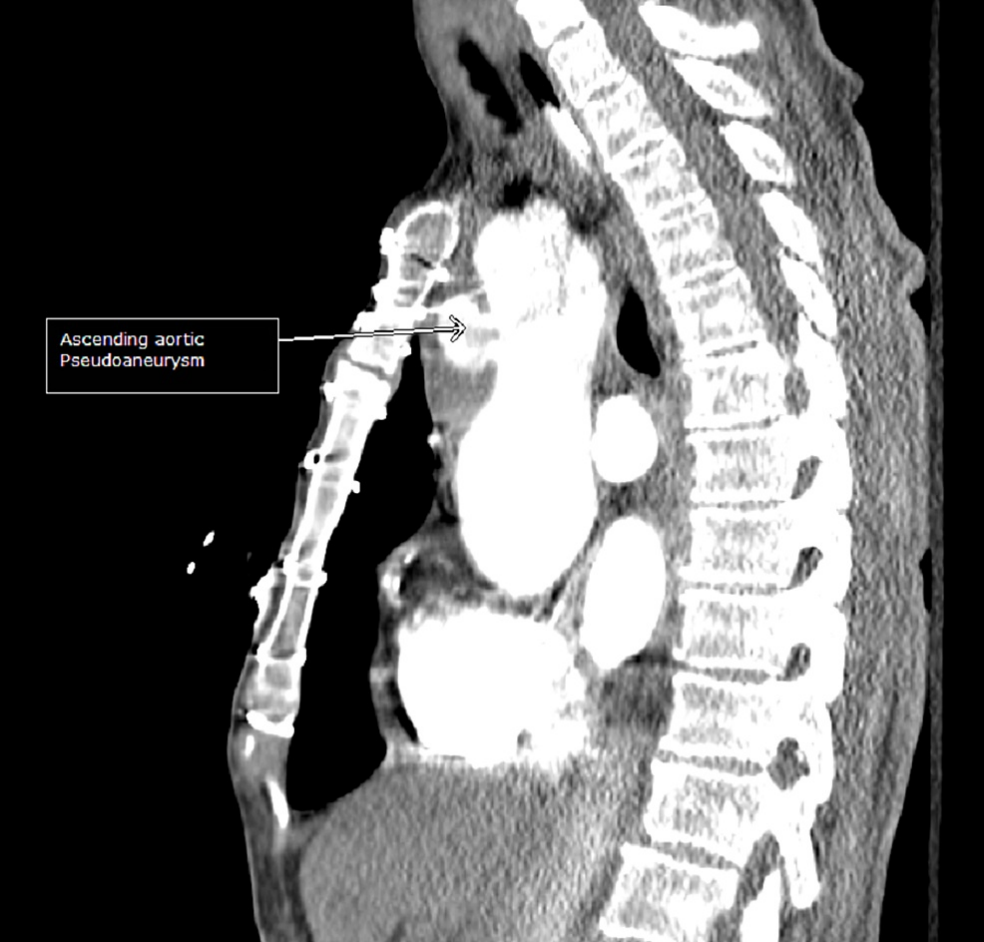
CT angiogram (sagittal view) demonstrating ascending aortic pseudoaneurysm.

On day 3 of hospitalization, the patient’s mental status deteriorated with a new GCS of 8 (E2V2M4). She was in a decorticate posture, tachycardic, with a heart rate in the 160s. Her oxygen saturation dropped to 80s on room air but improved to 95% on two liters of supplemental oxygen by nasal cannula. No repeat head imaging was done at this time. Subsequent repeat 12-lead ECG showed atrial fibrillation (Figure 6) and atrial flutter with 2:1 conduction. Repeat troponin was elevated (peak at 0.1 ng/ml), and brain natriuretic peptide was elevated at 139 Pg/ml. An elevated Wells score of 6 points, tachycardia, and hypoxia were concerning for an acute PE. A chest CT angiogram showed evidence of multiple bilateral sub-segmental PE (Figure 7), bilateral lower lung posterior segment consolidations (Figure 8), and a 5 cm ascending aortic lesion with 2 cm central ulceration highly concerning for a pseudoaneurysm (Figures 9-11). Despite the finding of PE, therapeutic anticoagulation was not started due to an increased risk of hemorrhagic conversion of the new ischemic stroke and potentially life-threatening hemorrhage from potential iatrogenic rupture of the incidental AAP. The patient was started on low-dose metoprolol tartrate for rate control and suppression of the high burden of PVCs noted during telemetry. The patient had a Revised Cardiac Risk Index (RCRI) score of 3 points with a 15% risk of 30-day mortality or adverse cardiovascular event following high-risk surgery. The risk versus benefits of repairing the pseudoaneurysm was discussed with the patient’s family. In view of her overall clinical state with a GCS of 8 and her elevated RCRI risk for high-risk surgery, the family and interdisciplinary care team concluded not to proceed with either surgical or endovascular repair of the AAP. Doppler ultrasound scans of both upper and lower extremities did not show any evidence of the presence of a DVT. However, an IVC filter was placed to mitigate the risk of recurrent thromboembolism from DVT originating in the lower extremities and pelvis, since the patient is now hemiplegic and bedbound.

On day 10, the patient's saturation continued to drop. She was intubated and placed on mechanical ventilation for acute hypoxic respiratory failure. An arterial blood gas test was done and showed a pH of 7.47, partial pressure of carbon dioxide (pCO2) 7.47, partial pressure of oxygen (pO2) 57 while 40% oxygen. A copious amount of respiratory secretion was noted in the endotracheal tube. This coincided with spiking temperatures of 101 F. Respiratory cultures, blood, and urine cultures were all negative. Oxygen concentration was increased to 80%. The patient was started on empiric vancomycin and meropenem per infectious disease recommendation. The fever eventually resolved. On day 13 of hospitalization, the patient pO2 significantly improved to 182. Her oxygen concentration was slowly weaned to 40%, after which she was successfully extubated and transitioned to supplemental oxygen by nasal cannula 4 liters per minute. The patient's mental status did improve with a new GCS of 10/15. The speech therapist conducted multiple unsuccessful swallow evaluation attempts. In view of the patient’s repeated failure of swallow evaluation and consolidation of her lower lung lobes noted on CT scan imaging, a percutaneous endoscopic gastrostomy tube was inserted for enteral feeding. The patient's vital signs remained stable on conservative management without anticoagulation for the PEs. She was discharged to a skilled nursing facility for continued physical therapy and assistance with daily living activities.

## Discussion

Acute aortic syndrome comprises aortic pathologies ranging from an aortic dissection, penetrating aortic/atherosclerotic ulcers, aortic aneurysm, aortic pseudoaneurysm, traumatic aortic transection, aneurysmal leak, and intramural hematoma [11]. These conditions have a rare incidence compared to other vascular pathologies; however, they increasingly add to all-cause/cardiac mortality prevalence and require urgent medical management [12]. AAPs have a 0.5% prevalence in patients who undergo complex cardiac surgeries and account for 3% of late mortality after CABG [13]. The aortic aneurysms and pseudoaneurysms also develop after a blunt/penetrating chest trauma or acceleration-deceleration injury from a motor vehicle crash or micro-injury to the abdominal aorta from a bullet fragment [14]. The iatrogenic aortic injuries due to endovascular procedures also add to the burden of AAP [15]. The open surgeries to the thoracic and abdominal cavities undertaken for non-vascular indications rarely trigger AAP [16]. However, to the best of our knowledge, iatrogenic AAPs due to trauma from CABG have a rare occurrence probably due to underdiagnosis and the absence of routine (post-CABG) screening. 

We reported an incidental 5 cm AAP discovered on the chest CT angiogram. The pseudoaneurysm was in proximity to the left internal mammary artery (LIMA) graft repair site created two years before the current hospitalization. The use of CT angiogram was based on its high specificity and sensitivity for AAP [12,15]. 

Our patient presented with multiple comorbidities including, acute ischemic stroke, paroxysmal atrial fibrillation (recorded via cardiac ECG), paroxysmal atrial flutter, AAP with central ulceration, multiple bilateral sub-segmental PEs, and a compromised lung reserve secondary to bilateral basal posterior lung consolidation. In addition, the patient had a high risk for perioperative mortality from surgical or endovascular graft repair of the AAP that restricted this intervention in the present scenario.

We presumed that the paroxysmal atrial fibrillation and flutter probably triggered thrombi formation in the left atria. These were embolized to the systemic circulation, leading to multiple ischemic strokes involving different cerebral arterial territories [17]. The possible mechanism for the development of acute PEs correlates with the right atrial thrombi. The clinical studies indicate a 10% attribution of intracardiac thrombi to PE [18]. The thrombi rarely develop in the right atrium but increase the risk of multiple PEs [19]. Transthoracic echocardiogram (TTE), in the present case, did not show any intramural thrombus within the heart chambers. We could not perform a transesophageal echocardiogram (TEE) despite its higher sensitivity than TTE ( 35% vs 11%) [20] in diagnosing intracardiac thrombus, patent foramen ovale, and intracardiac masses since the patient’s family was more concerned with restricting the invasive procedures to the possible extent.

The most challenging issue with our patient was to select a minimally invasive approach based on the myriad of clinical complications. The exclusion of thrombolytic therapy in the present case was due to the high risk of fatal iatrogenic rupture of the thoracic AAP [21]. The patient could have received thrombolytics only after presenting with a definitive history of new stroke symptoms within 3-4.5 hours of their onset and a negative intracranial hemorrhage finding from the CT head [22]. 

The clinical practice guidelines do not recommend routine screening for aortic pathology in patients with ischemic stroke due to the rare occurrence (0.5%) of aortic aneurysm or AAP after CABG [23]. The history of multiple acute PEs and paroxysmal atrial fibrillation/flutter with a high CHA2DS2-VASc score (i.e., 6 points) substantiated the anticoagulation treatment in the present case [24]. However, the shared decision-making between the patient’s family and the interdisciplinary team excluded the anticoagulation treatment due to the markedly higher risk of AAP rupture. 

CHA2DS2-VASc is an independent predictor of mortality and thromboembolic events in patients with/without atrial flutter and atrial fibrillation [25]. It also guides the antithrombotic therapies based on the risk of cardiovascular complications. These treatments include dose-adjusted therapy with vitamin K antagonists and oral anticoagulation [26]. Since our patient was not a candidate for anticoagulation, placement of a WATCHMAN device into the left atrial appendage was considered but not performed based on the negative intracardiac thrombi finding from TTE [27]. We subsequently placed an IVC filter to minimize the risk of PE, triggered by clots in the deep veins of the pelvis and lower extremities [28]. The AAP repair was not undertaken due to the increased risk of cardiovascular events and mortality.

## Conclusions

The American College of Cardiology and American Heart Association guidelines do not recommend screening for aortic pathology before thrombolytic therapy or anticoagulation in asymptomatic patients with acute ischemic stroke and a history of CABG. In addition, the high cost of CT imaging and the potential radiation exposure also challenge the routine screening for aortic pathologies in asymptomatic post-CABG patients who require anticoagulation or thrombolytics for other indications. The screening is necessitated in scenarios with a high suspicion of aortic pathology and associated symptoms on a case-to-case basis.
